# Use of a virtual world computer environment for international distance education: lessons from a pilot project using Second Life

**DOI:** 10.1186/1472-6920-14-36

**Published:** 2014-02-21

**Authors:** Marloes Schoonheim, Robin Heyden, John M Wiecha

**Affiliations:** 1 Geneva Foundation for Medical Education and Research, Villa Gran-Montfleury, Chemin du Grand-Montfluery 48, 1290 Versoix, Switzerland; 2 Heyden Ty, 2500 San Jose Avenue, Alameda, CA 94501, USA; 3 Office of Medical Education, Boston University School of Medicine, 72 East Concord St., B2900, Boston, MA 02118-2518, USA

**Keywords:** Medical education, Continuing medical education, Computer-assisted instruction, Computer aided instruction, Distance education, Computer simulation

## Abstract

Virtual worlds (VWs), in which participants navigate as avatars through three-dimensional, computer-generated, realistic-looking environments, are emerging as important new technologies for distance health education. However, there is relatively little documented experience using VWs for international healthcare training. The Geneva Foundation for Medical Education and Research (GFMER) conducted a VW training for healthcare professionals enrolled in a GFMER training course. This paper describes the development, delivery, and results of a pilot project undertaken to explore the potential of VWs as an environment for distance healthcare education for an international audience that has generally limited access to conventionally delivered education.

## Background

Virtual worlds (VWs) are immersive, online environments where the imaginary meets the real to promote shared experiences and the exchange of ideas and information. They are online spaces, accessed through a computer graphical user interface, where all that is perceived takes place in real time.

**Figure 1:**
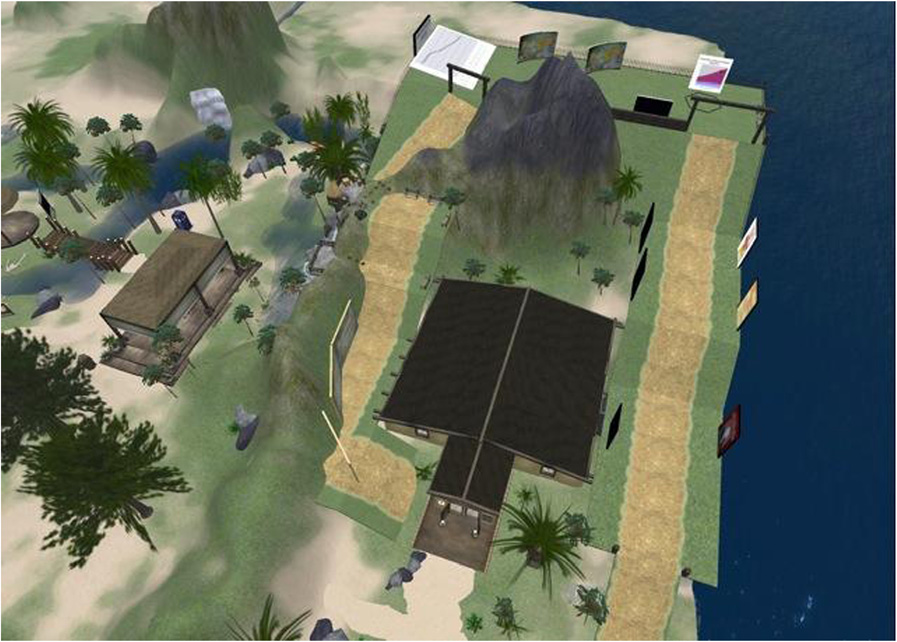
**The virtual world of Second Life.** This aerial photo shows the virtual location of the WHO/GFMER/BU island where the educational event took place.

VWs graphically represent physical space in three dimensions and support high levels of social networking and interaction [Figure 1]. Participants enter VWs as “avatars” and can explore, meet other avatars, communicate and socialize, participate in individual and group activities, create objects, and learn from designed experiences [1].

Second Life (SL) is a virtual world created (in 2003) and maintained by Linden Lab (San Francisco, California). With approximately 36 million registered users, it is among the most popular VW platforms [2].

VWs are experiential environments with tremendous potential for effective teaching and learning [3-5]. Immersive, realistic, and engaging online events can bring high quality medical education to healthcare workers in remote locations [6-8].

The authors collaborated to adapt one session of their online course, jointly organized by the Geneva Foundation for Medical Education and Research (GFMER) and the World Health Organization (WHO), for a pilot implementation in Second Life (SL). The purpose of this pilot project was to explore the potential of VWs as an alternative medium for distance healthcare education in developing countries.

GFMER and WHO offer their annual online course “From Research to Practice: Training Course in Sexual and Reproductive Health Research” to hundreds of participants in more than forty, mainly developing, countries. The professional background of the participants is diverse: many are physicians but the course also caters to nurses and other healthcare professionals.

Now in its third year, the online course is a very successful training package especially for those in developing countries where sexual and reproductive health challenges are considerable. In most low-income nations, access to conventional health care education is generally limited: professionals may lack financial resources and live and work in remote areas with poor infrastructure or in a conflict zone. Internet coverage in developing countries, however, has increased considerably over the past few years. Distance learning is an important way to offer health care professionals in developing countries the opportunity to increase their clinical and research skills.

Course staff use social media and discussion boards (Google+, Facebook, Twitter) to interact with learners and provide a platform for learners to communicate. These communication tools, however, are limited. They are two-dimensional, not immersive, and limited in the way educational information can be designed.

This pilot proposed to investigate the potential of using VWs in this course and to assess the event’s impact and the learners’ attitudes following the event.

This article will describe our pilot’s instructional design, the event itself, the participants’ response to the event, and provide a summary of lessons learned for future virtual world learning developers.

## Methods

We selected “Population Control: Past Policies and Future Challenges,” taught by Dr. Marloes Schoonheim, research fellow of GFMER, for our VW pilot session. Dr. Schoonheim and the VW producers designed a learning environment and instructional design appropriately adapting the content typically included in this session of “Training Course in Sexual and Reproductive Health Research”.

The VW session was held on May 3, 2012. Learners joined the event on their computers in one of three ways:

1) Synchronously in Second Life as an “avatar”

2) Synchronously watching a live broadcast of the event in their browser

3) Asynchronously viewing a video capture of the event.

These three alternatives provided options to accommodate all learners’ available computer power and Internet bandwidth.

The live, VW session was 60-minutes in length. Participants were learners enrolled in “Training Course in Sexual and Reproductive Health Research”. Those who chose to participate as avatars were offered basic SL training through a combination of web-based videos, guides, and scheduled “office-hours” for one-on-one guidance.

The session’s content included an overview of the growth of the world’s population, a recap of historical views on population concerns, and other relevant topics in population control.

**Figure 2:**
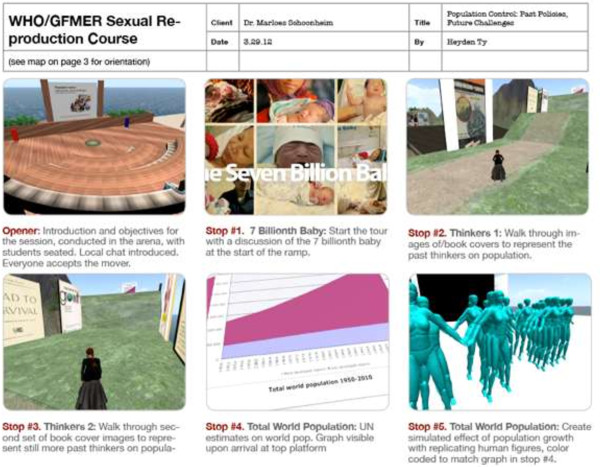
**Storyboard.** A sample page from the project storyboard that describes activities at each learning station.

The instructional design for the session was adapted from Dr. Schoonheim’s existing course. We created a session storyboard [Figure 2] that integrated lecture, interactive experiences and virtual world special effects to make effective use of the medium.

**Figure 3:**
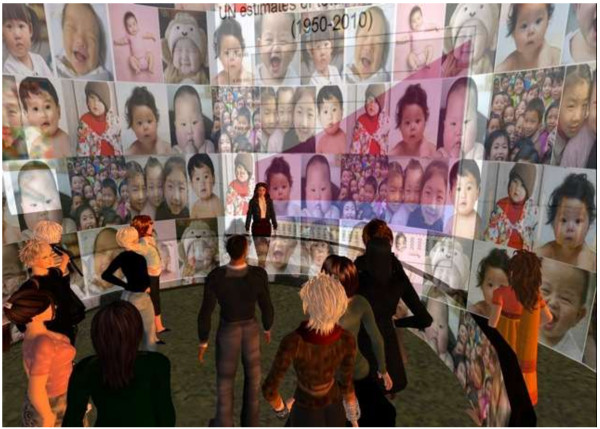
**Example display.** This learning station displayed a very large rotating screen of racially diverse baby photographs to illustrate population dynamics.

Next, we began to design the virtual location in which the session would take place. Mioncha and Hardy [9] make clear that learning space design impacts the learners’ processing of information. Among their principles, they point out the importance of orientation upon arrival, clear navigational signs and symbols, easy to follow pathways and well-structured, easily navigable spaces. With these principles in mind, we created an open air seating arrangement and a series of easily navigable ramps, with a well-worn and obvious dirt path to follow. The ramp formed a sort of gallery, with learning stops marked along the path with a large visual (e.g. a photograph, map, or book cover). Interactive methods, such as a rotating graphic cylinder simulating the effect of an intensely populated area, focused learner’s attention. World maps helped participants locate countries referenced in the discussion [Figure 3].

The development team conducted three rehearsals prior to the live event, resolving technical problems and session transitions.

The session began and ended in the open-air seating arrangement. As the avatars appeared they chatted with each other through typed local chat or tried their microphones to speak. The producers helped to resolve sound problems and answered questions while students practiced walking, changing their view/perspective, sitting, and flying.

**Figure 4:**
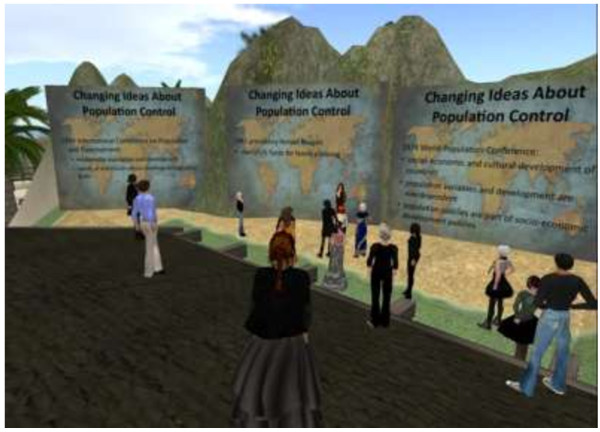
**Learning station.** Dr. Schoonheim invited questions and discussion at each learning station.

Dr. Schoonheim warmly welcomed the learners and explained that this would be an experiment for all of us and she was clear to invite participation and questions. The student avatars then followed her onto the ramp and proceeded along the path to the series of learning stations containing graphics. At each graphic Dr. Schoonheim stopped to provide explanations, raised questions, and invited discussion [Figure 4]. The need to physically move as the course progressed kept everyone alert and it was easy for Dr. Schoonheim to see that the students were *literally and figuratively following her*. As the hour progressed, students became increasingly engaged, asked clarifying questions, and offered comments. Here is a sampling of questions asked during the session in the local chat: “Is contraception publically available in China?”; “Are the religious forces as strong and active as they are in the U.S.?”; “We have to understand the belief that Family Planning and Population issues are mostly concerns of women which makes the service more women-focused.”; “In Nigeria, the fertility rate is 5.7”; “How about the influence of technology? Does shift work and work force have bearing on this?”.

The easy exchange of ideas with people from all over the globe gave the course a uniquely collaborative feeling. Eventually, arriving back at the amphitheater, Dr. Schoonheim delivered final comments and hosted a brief question/answer session.

Dr. Schoonheim was the only person speaking during the session. Avatar learners, as well as those listening to the Livestream broadcast, could hear her voice. Second Life participants engaged with Dr. Schoonheim and each other by typing in SL’s local text chat. Healthcare professionals participating through the live broadcast were invited to comment or ask questions through an online chat module the producers bridged into Second Life. A short event video is posted online (https://vimeo.com/45091079).

## Results

Nineteen avatars attended the virtual world session, 11 of whom were learners, and forty viewers participated through the Livestream session.

Following the event, all 11 learners completed a nine-question survey. Table 1 summarizes the survey data. Respondents were overwhelmingly positive about the virtual world learning environment. They all agreed that the virtual world environment is an improvement over other common modes of online learning such as webinars, and few disagreed with the statement that the virtual world is an improvement over a face-to-face setting. Respondents largely felt that course objectives were fully met.

**Table 1:** Post-event survey results

***Rate your level of agreement with the following statements:***	**Strongly agree N (%)**	**Tend to agree N (%)**	**Neither agree nor disagree N (%)**	**Tend to disagree N (%)**	**Strongly disagree N (%)**
The virtual world of Second Life is an effective environment in which to learn.	10 (90.9)	1 (9.1)	0	0	0
Learning in a virtual world is an improvement over learning in an online course or webinar.	7 (63.6)	4 (36.4)	0	0	0
Learning in the virtual world is an improvement over learning in a face-to-face setting.	2 (18.2)	1 (9.1)	6 (54.5)	2 (18.2)	0
***To what degree were the following objectives met?***	**Fully met**	**Met mostly**	**Partially met**	**Did not meet**	
Discuss how population control has been influenced by politics and economics.	8 (72.7)	3 (27.3)	0	0	
Explain why family planning is not a simple solution for overpopulation.	8 (72.7)	3 (27.3)	0	0	
Distinguish between population control on a theoretical level and the way it was implemented in China and India.	8 (72.7)	3 (27.3)	0	0	

### Dr. Schoonheim’s impressions as a presenter in a virtual world: a first person perspective

The opportunity to present in the virtual world was an amazing experience for me. Although I’ve given many face-to-face and webinar presentations on this topic – attitudes of national and international governments to controlling population growth – the presentation in Second Life added considerably to the learning experience.

First, the virtual world venue is immersive and interactive. This means the audience doesn’t just hear me speak, but is able to walk around and virtually touch the presentation. This really helped to make important points clear and indelible. For instance, the 1950s and 60s fear that population would outgrow increases in the means of subsistence. By walking between rows of huge “Population Boom” book covers, we were able to convey the alarm these publications induced. I found it easier to keep the audience’s attention on an otherwise dull graph, depicting population growth, when we invited them to walk across the graph, hover over it and, literally, experience the data.

A presentation in Second Life adds to the learning experience by offering a fun and informal atmosphere in an extraordinary setting. I have never given a presentation walking on an island dirt path, in (virtual) open air, surrounded by trees and the sea. I’m quite certain our audience (consisting of health care professionals from developing countries) hadn’t either. In addition all participants dressed their own avatars using the seemingly endless variations possible in Second Life, sometimes selecting a pretty wild appearance. The result was a vibrant, informal atmosphere and an authentic community feeling among participants. Perhaps it was that community feeling that encouraged the audience to ask questions and raise issues; something I notice is lacking in face-to-face presentations or more formal settings, particularly when the audience is unfamiliar with demography.

My Geneva Foundation for Medical Education and Research colleagues and I are convinced that distance learning offers health professionals in developing countries the necessary access to quality education in the field of sexual and reproductive health. However, we also see high dropout rates in online courses. Online courses are typically given as webinars, which can be boring, can increase a feeling of isolation, and often participants are inclined to combine online lectures with other activities. A learning event in Second Life with active participation and favorable group dynamics can address these problems although moderators must keep the audience occupied and avoid silent times. The amazing team I was privileged to work with made sure we avoided those pitfalls by planning my presentation and rehearsing with me. With their help, it was easy to get used to moving my avatar while presenting information by speech, slides and keyboard.

I still hear from participants who regularly refer to the event and I can see it made a strong impression on them. They remember much of the population control information from the session, thanks to the effective, innovative and fun learning experience Second Life offers.

## Discussion

The survey results confirm that the application of the virtual world Second Life to this learning activity was successful in many respects (Table 1). All participants agreed that the virtual world was an effective learning environment, and was an improvement over webinar-like designs, and over a quarter endorsed that the VW learning was an improvement over a face-to-face environment.

The learners gave high ratings to the session’s effectiveness on all learning goals (Table 1) and cited many advantages to this form of learning. They were engaged, asked informed questions, and chatted with each other about the session content and its significance.

This pilot generated valuable lessons, which can be applied to future virtual world learning events:

– Rehearsal time is mandatory. With multiple rehearsals, the facilitator becomes increasingly comfortable with the participation options (as well as the content of their material) and the team achieves increased problem solving fluidity.

– We have learned through delivering many such virtual world events that it is essential for the facilitator to be comfortable in the virtual world. In our experience the participants take their cue from the facilitator. If she is relaxed, calm, and resourceful, they will be as well.

– Strive for an effective balance between didactic presentation and interactivity. Learner interactivity improves engagement and content retention, but some content is better delivered straight.

– Developing learning environments in virtual worlds can be expensive. We controlled our costs when we constructed a flexible, open-air location that could be re-used for multiple events, trained SL producers and used the same people repeatedly, and retained the services of a video producer who could capture video footage and manage the Livestream broadcast simultaneously.

– Establish a plan for fielding participant questions. So many good questions reflect interest and engagement, but they must be efficiently managed.

– Technical challenges are inevitable. There are numerous technical challenges to address when conducting such an event. The internet bandwidth requirements for virtual world platforms and occasionally encountered firewalls make access impossible for some. It is wise to have an alternative attendance method for those participants. In our case, we offered the Livestream (browser-based) channel as well as an archived recording of the event.

The most frequently encountered technical problem in Second Life is sound. Since our event depended on the participants’ ability to hear the speaker, we retained experienced SL staff to diagnose and solve sound problems as they arose. We encouraged all participants to arrive early and followed a practiced protocol for resolving sound problems (headset, to computer controls, to Second Life sound preferences).

### Limitations

Several potential limitations are notable including a small sample size of participants using the VW environment to access and then evaluate the course experience, and potential bias of evaluation responses based on self-selection into the VW group. In addition, the pool of participants was drawn from a larger group all of whom were enrolled in an online course, thus likely to be more experienced and comfortable with online learning environments generally. However, our previous work has shown that even individuals completely unfamiliar with a VW, and even with quite low educational levels, are able to effectively master a VW for educational purposes and rate it favorably [10].

## Conclusion

For participants in this pilot project, the virtual computer-based world was an effective learning environment, and was an improvement over more conventional online learning environments like webinars. Although some participants even felt the VW was an improvement over a face-to-face strategy, most were either neutral or disagreed, suggesting as expected that face-to-face delivery remains the gold standard. Students were participatory, and although there were technical problems, these were not insurmountable and were addressed before or during the session for most participants.

While conventionally designed distance learning and online courses are good solutions for geographically dispersed students, learners in those courses may feel isolated and disconnected from their peers. Yet logistics and expense often make it impossible to bring course participants face-to-face.

VW learning was an excellent solution for these challenges faced by GFMER and WHO in this pilot. VW learners can gather in an immersive space for an authentic feeling of collaboration and exchange without the need for passports, visas, or travel expenses of the real world. While more traditional distance education tools can also reduce geographical burdens, their lack of immersiveness and presence renders them less engaging and potentially less effective when compared with live, in-person events. The VW entices learners to leave their familiar face-to-face or online learning environment and travel to a new, virtual destination to meet other learners from different backgrounds and locations. Active participation required at this new, virtual location enhances the learning experience. As such VW learning can promote international exchange of expertise, theory, and practice – particularly valuable for healthcare learners in underserved countries. Although lack of bandwidth could be an obstacle for participation for some learners, this pilot successfully attracted participants from resource-poor settings by providing them with access options tailored to their particular bandwidth considerations.

A virtual world setting can accommodate distance learners at many levels of participation. Special virtual world text, visuals, and voice provide alternative paths toward mastering information. Learners can listen to the presentation, focus on the visuals, exchange information with their colleagues, and interact with the exhibits. They can also return to the unique environment at a later time to continue to learn or share the archived video of the live event with others.

## Competing interests

All the authors declare they have no competing interests.

## Authors’ contributions

As the course instructor, MMS was solely responsible for the intellectual content of the event. MMS researched and created the content slides, helped to design the learning stations, and drafted the manuscript. RH was the instructional designer, Second Life builder, and drafted the manuscript. JW obtained the original funding, guided the overall plan, and helped to draft the manuscript. All authors read and approved the final manuscript.

## Authors’ information

MS has a Ph D in historical demography. She is a researcher and develops distance learning courses for the Geneva Foundation for Medical Education and Research in Switzerland.

RH is an education consultant in Alameda, California. She plans, designs, and constructs online learning experiences, specializing in the use of social media and virtual worlds for teaching and learning.

JW is an Assistant Dean for Academic Affairs at the Boston University School of Medicine, and a family physician. He has a particular interest in the design and evaluation of innovative internet-based health education methods.

## Pre-publication history

The pre-publication history for this paper can be accessed here:

http://www.biomedcentral.com/1472-6920/14/36/prepub
